# Level of Knowledge and Needs on Fertility Preservation in Reproductive-Aged Male Patients with Cancer

**DOI:** 10.1007/s13187-018-1467-9

**Published:** 2019-01-07

**Authors:** Hanfeng Zhang, Guorong Wang, Maoqiu Cao, Li Yin, Yan Xing, Jing Wang, Jing Yang, Jian Zhang

**Affiliations:** 1 Department of Radiation Oncology, Sichuan Cancer Hospital & Institute, Sichuan Cancer Center, School of Medicine, University of Electronic Science and Technology of China, 55, 4th Section of Renmin South Road, Chengdu, 610040 Sichuan Province People’s Republic of China; 2 Department of Nursing, Sichuan Cancer Hospital & Institute, Sichuan Cancer Center, School of Medicine, University of Electronic Science and Technology of China, 55, 4th Section of Renmin South Road, Chengdu, 610040 Sichuan Province People’s Republic of China

**Keywords:** Fertility preservation, Cancer, Patient preferences

## Abstract

There is a growing concern about the fertility preservation (FP) for cancer patients of childbearing age. This study is the first in China to survey men with cancer, of reproductive age, regarding their knowledge of FP and their related needs. A 12-item cross-sectional survey was conducted of 332 male patients. The score for knowledge of FP was 3.5 ± 0.67, of a possible score of 8. Only 10.6% of the subjects had chosen to preserve fertility before treatments, but during therapy 68.7% wanted more information about FP. Younger patients were more likely have more knowledge concerning FP than older patients (odds ratio [OR] 0.86). The decision to make arrangements for FP before treatments was heavily influenced by being young and without children (OR, 0.78; OR, 0.11). Male cancer patients of reproductive age had limited knowledge of FP, and the majority was disinclined to make FP arrangements before therapy in China. Therefore, male cancer survivors should be well informed about FP soon after diagnosis and programs should be considered to improve the FP-related knowledge of male cancer survivors. We suggest that an assessment of patients’ understanding of FP issues, before treatment, should be standard in clinical work.

## Introduction

According to the National Coalition for Cancer Survivorship, an individual is considered a cancer survivor from the time of diagnosis until death from any cause [1]. This definition of a cancer survivor is now widely recognized by many authors [2]. With advances in cancer treatment, the survival rates of cancer patients have considerably improved, and therefore increasing attention has been turned to the issue of long-term quality of life of cancer survivors.

Cancer, or treatments for cancer, can affect patients’ ability to have children, and fertility is an important aspect of quality of life for cancer survivors of reproductive age [3, 4]. Reports indicate that cancer survivors with fertility options struggle against the cancer more actively [5], whereas infertile patients experience great distress and grief [6, 7]. In fact, many cancer survivors report a strong desire to maintain fertility and prefer to have their own biologic children [8–10].

Thanks to improvements in assisted reproductive technology, many cancer patients have the option to preserve their fertility. For men, sperm cryopreservation is a standard strategy for fertility preservation (FP) recommended by the American Society of Clinical Oncology and the European Society for Medical Oncology [3, 11]. Other strategies, such as testicular tissue freezing and testicular sperm extraction, are still at the stage of experimentation [12].

International guidelines suggest that oncology physicians should provide sufficient information regarding FP to cancer patients of childbearing age [11, 13]. Although many studies have investigated the knowledge and attitudes of oncology physicians regarding FP [14, 15], there are very few published studies concerning patients’ actual understanding of FP or their related needs. Given the importance of FP to patients, and the success rate of sperm cryopreservation in male cancer survivors, this study explored the FP-related knowledge and needs of male cancer patients of reproductive age.

## Methods

The Ethics Committee of Sichuan Cancer Hospital and Institute approved the study.

All participants provided written informed consent.

## Participants and Setting

A cross-sectional survey was conducted from July 2017 to June 2018 at Sichuan Cancer Hospital and Institute, to evaluate male cancer patients’ knowledge of FP and their needs related to FP. The inclusion criteria of the study were male aged 18–45 years, on initial admission to the hospital, and undergoing or already finished treatments that threaten fertility. Potential subjects with any of the following were excluded from this study received anticancer treatments in other hospitals, declined to participate in the study, or did not complete the questionnaire.

## Measures

The 12-item questionnaire was developed by a multidisciplinary team of researchers, oncology physicians, and reproductive experts who were all familiar with FP. The items were reviewed and revised by survey experts. The modified questionnaire then was piloted with a small group of male patients with cancer to test its validity and acceptability. The finalized questionnaire, containing three sections (demographic information, knowledge of FP, and FP-related needs), was used in this study. Researchers had access to information that could identify individual participants during or after data collection.

### Individual Demographic Information

The demographic data included patients’ age, ethnicity, career, education, diagnosis, stage of cancer, marriage status, number of children, monthly income of family, and type of insurance.

### Knowledge of FP

There were eight statements on the questionnaire to evaluate knowledge of FP. Among the eight, there were three questions to assess subjects’ knowledge of an association between cancer treatments and decline in fertility (e.g., “Cancer treatment can affect fertility?”). In addition, five questions evaluated basic knowledge of FP: FP methods, organizations in Sichuan Province, and issues related to storing (banking) sperm.

One point was award for each correct answer or positive statement (“I Know”); otherwise, the score was zero. The overall possible knowledge score was 8 points.

### Patient’s FP-Related Needs

One item was used to assess if patients had chosen the sperm bank to preserve their fertility before treatments. Three items were designed to understand patients’ needs for getting information about FP. A final free text box was added to allow patients to clarify their reasons for refusing to preserve fertility before treatments and to state their preferred ways to receive FP-related information.

## Statistical Analysis

Data were analyzed using SPSS 19.0 software. All *P* values are two-sided, with a statistical significance level set at *P* < 0.05. Frequencies and proportions were summarized for demographic characteristics and each survey item. Using binary logistic regression, odds ratios (ORs) and their 95% confidence intervals (CIs) were estimated to compare demographic characteristics with knowledge score or FP-related needs. Questionnaires with missing data were excluded from the statistical analyses.

## Results

### Response Rate

A total of 360 patients participated in the survey from July 2017 to June 2018. Among these, 332 patients (92.2%) were eligible. The 332 valid questionnaires were collected and no missing data was found.

### Demographic Characteristics

The mean age of the subjects was 35.5 years (Table 1). Of our overall sample, the majority of respondents was married, of Han ethnicity, and had stage III colorectal cancer. Most patients had a monthly family income of $721–1150, and were covered by city-level medical insurance. The average number of children among these participants was one child.

**Table 1:** Demographics of participants

Subjects, *n*		332
Age, years		35.5 ± 6.2
Career	Permanent job	176 (53)
	Freelancer	32 (9.6)
	Farmer	100 (30.1)
	Others (e.g., students)	24 (7.3)
Education	≤ Junior high school	128 (38.6)
	Senior high school	128 (38.6)
	≥ Bachelor’s degree	76 (22.8)
Marital status	Married	268 (80.7)
	Single	36 (10.8)
	Divorced	28 (8.4)
Children, *n*		1.0 ± 0.01
Ethnicity	Zang	16 (4.8)
	Han	316 (95.2)
Family income, per month, $	145–570	64 (19.3)
	571–720	48 (14.5)
	721–1150	120 (36.1)
	≥ 1150	100 (30.1)
Medical insurance	Rural	112 (33.7)
	City	172 (51.8)
	Provincial	48 (14.5)
Type of cancer	Colorectal	148 (44.6)
	Malignant lymphoma	88 (26.5)
	Testicular	20 (6)
	Prostate	76 (22.9)
Cancer stage	I	24 (7.2)
	II	100 (30.1)
	III	172 (51.8)
	IV	36 (10.8)

Reported as mean ± standard deviation or *n* (%), or as indicated

### Knowledge of FP

The mean score of respondents for knowledge of FP was 3.5 ± 0.67 points, of a possible total of 8 points. Among these participants, 77.8% of them were aware that cancer treatment could damage fertility, while 63.9 and 80.7%, respectively, were unfamiliar with FP methods or FP organizations in Sichuan Province. More than 71.1% of the men were unclear about the function of a sperm bank, and 97.8% were not sure about how long they should wait to conceive after cancer treatment. About 27.7% of participants were concerned that they might pass a cancer gene to future children.

### FP Requirements

In this study, only 10.6% of the men chose to use a sperm bank to preserve fertility before cancer treatments. The reasons for declining FP were the following: age (20.8%), financial burden (10.1%), previous children (40.3%), cancer treatment as a priority (56.6%), and fear about the influence of genetic factors on children (6.1%). About 20.3% of male patients with cancer stated that they did not get FP-related information from their treating physicians. Nearly 68.7% of patients wanted more information about FP during treatments. Patients preferred to receive information through oral discussion with health care providers (92.9%), booklets (56.1%), and the Internet (45.6%).

### Association Between Demographics and FP Knowledge or FP Needs

Binary logistic regression was used to compare the demographic factors with the knowledge scores (< 4, and ≥ 4) and desire for FP (need, not need; Table 2 and Table 3). Younger male patients with cancer were more likely to be more knowledgeable regarding FP (OR 0.86, 95% CI 0.75–0.98). Patients with monthly family incomes of $571–720 (OR, 0.03) and $721–1150 (OR, 0.23) had lower knowledge scores than did patients with monthly family incomes of > $1150. Regarding the need for FP, younger men (OR, 0.78) and patients with fewer children (OR, 0.11) were more prone to make FP arrangements.

**Table 2:** Logistic regression analysis of factors associated with FP-related knowledge

		OR (95% CI)	*P*
Age		0.86 (0.75–0.98)	0.03
Number of children		0.56 (0.17–1.81)	0.33
Family income, per month, $	145–570	0.01 (0.01–1.13)	0.97
	571–720	0.03 (0.02–0.42)	0.01
	721–1150	0.23 (0.06–0.92)	0.04
	≥ 1150	–	–
Cancer stage	I	–	–
	II	1.58 (0.14–18.0)	0.71
	III	0.95 (0.10–10.1)	0.97
	IV	1.86 (0.10–34.37)	0.68

**Table 3:** Logistic regression analysis of factors associated with patients’ desire for sperm preservation

		OR (95% CI)	*P*
Age		0.78 (0.02–1.61)	0.04
Number of children		0.11 (0.01–0.84)	0.03
Family income, per month, $	145–570	0.34 (0.01–10.51)	0.54
	571–720	2.11 (0.05–84.23)	0.69
	721–1150	0.71 (0.03–17.30)	0.83
	≥ 1150	–	–
Stage of cancer	I	–	–
	II	0.29 (0.02–3.69)	0.34
	III	0.09 (0.03–3.04)	0.18
	IV	0.04 (0.01–1.53)	0.08
Knowledge score	< 4	2.74 (0.16–47.66)	0.49
	≥ 4	–	–

## Discussion

To our knowledge, this is the first survey of reproductive-aged male patients with cancer regarding their knowledge and desire for FP. A previous study in 2002 suggested that sperm banking should be offered as an option to all men at risk of infertility, before cancer treatments began [16]. Subsequently, in 2006, international guidelines recommended sperm cryopreservation for male cancer patients who had the desire to father a child [17]. Considering the advances in communication technology since then, it is logical to suppose that reproductive-aged men with cancer may have full access to information related to FP. Nevertheless, in this study, the knowledge score of male patients with cancer was only 3.5, out of a possible total of 8 points, which was lower than that of female patients with cancer in a previous study [18]. Although 79.7% of the men in the present study stated that they were informed on fertility issues, more than 70% were unclear about sperm banking or how long they should wait to conceive after cancer treatment. It is confusing that male patients had been informed about FP, but still had a low knowledge scores.

The reasons for this discrepancy may be related to the nature and timing of communications between male patients and their treating physicians. Previous studies revealed that before treatment many cancer patients focus solely on survival, and are not so concerned with the effects of treatment on future fertility [19]. Consistent with this, in the present study, nearly 60% of the men considered cancer treatment as the main priority, and therefore may have ignored the option and information they received regarding FP. In addition, the mean age of the men in this study was 35.5 years, and most patients had a monthly family income of $721–1150. The slightly older age and limited economic status were associated with low knowledge level (OR = 0.86; 0.03; 0.23).

Some oncology physicians are reluctant to discuss fertility issues with their patients [20, 21], and this may also have contributed to the low knowledge of the men in the present study. Current data suggest that more studies should be conducted to find out why oncologists do not adhere to the guidelines regarding FP, which have been recommended for more than 10 years. In addition, after a discussion of FP issues with the patient, the treating physicians should request feedback from them to assess their understanding of what they have been informed. More programs should be developed to improve FP-related education for male patients with cancer and their families.

Although sperm cryopreservation is easily accessible and widely available, only 10.6% of our patients used this option before their cancer treatments, which is much lower than the rates reported previously [22, 23]. In addition, in the present study younger male patients and those with no child were more likely to make plans for FP (OR, 0.78; OR, 0.11). The average age of our patients was 35.5 years, and the average number of children was one child. Thus, 20.8 and 40.3% of the male participants, respectively, gave up the option to maintain fertility because of age or current parenthood. Other factors that influenced the decision to select sperm cryopreservation were the patients’ economic situation, concerns regarding genetics, and the priority of treatment.

With regard to the expense of storage in sperm cryopreservation, some authors have suggested that a portion of the cost should be covered by medical insurance, to lighten the economic burden of cancer survivors [23]. However, this change in insurance policies may take a long time to achieve. In addition, there is no empirical evidence that cancer confers health risks to future offspring. We suggest that male cancer survivors be treated by a multidisciplinary team of oncology physicians, reproductive endocrinologist, and oncology nurses who are familiar with FP.

In this study, male cancer survivors were little concerned about their fertility at the time of cancer diagnosis, but more than half became interested in FP during cancer treatment. This may be because at the time of diagnosis the principle concern was survival and treatments, while some patients began to think about fertility issues during treatments.

In traditional Chinese culture, continuing the family line is important. In 2015, the government of China approved a new child policy, in which each couple is allowed to have two children. This new policy provides every Chinese person, including cancer patients, permission to have another baby. Therefore, it should be guaranteed that every patient of childbearing age, whether or not he or she already has a child, have access to sufficient information about FP, to be provided by their treating physicians. Thus, the best way to avoid medical disputes is for patients to be well informed of their FP options, prior to cancer treatments that may compromise their fertility [24].

Previous studies have shown that some young cancer patients are reluctant to discuss fertility issues in the presence of their parents [25]. In the present study, we surveyed patients regarding their favored way to receive FP-related information. Patients preferred to receive information from health care providers, booklets, and the Internet, at rates of 92.9%, 56.1%, and 45.6%, respectively. These results suggest ways of offering FP information in clinical practice. In addition, programs should be designed to help both health care providers and cancer patients feel comfortable discussing FP.

### Limitations of the Study

This study is the first to report the present knowledge and needs of male cancer survivors of childbearing age concerning FP. However, the sample size may not be large enough to represent the entire population of male cancer survivors in China. Furthermore, only 4.8% of the participants were of Zang ethnicity (Tibetan people). Further studies are warranted, with study populations of more diverse ethnic backgrounds.

## Conclusions

Our survey provides direct evidence that knowledge about FP in male cancer survivors of reproductive age is generally poor, and before cancer treatment they are unlikely to take steps to ensure their future ability to have children. Nevertheless, during treatments some patients were interested in obtaining more information regarding FP. Therefore, male cancer survivors should be well informed about FP soon after diagnosis, in effective and comfortable ways. We suggest that an assessment of patients’ understanding of FP issues, before treatment, should be standard in clinical work. In addition, programs should be considered to improve the FP-related knowledge of male cancer survivors.
